# Melatonin-Induced Cytoskeleton Reorganization Leads to Inhibition of Melanoma Cancer Cell Proliferation

**DOI:** 10.3390/ijms21020548

**Published:** 2020-01-15

**Authors:** Alejandro Alvarez-Artime, Rafael Cernuda-Cernuda, Francisco-Artime-Naveda, Vanesa Cepas, Pedro Gonzalez-Menendez, Sheila Fernadez-Vega, Isabel Quiros-Gonzalez, Rosa M. Sainz, Juan C. Mayo

**Affiliations:** 1 Departamento de Morfología y Biología Celular, Redox Biology Group, Instituto Universitario Oncológico del Principado de Asturias (IUOPA), School of Medicine, University of Oviedo, C/Julian Claveria 6, 33006 Oviedo, Spain; alejandroalvarezartime@gmail.com (A.A.-A.); rcernuda@uniovi.es (R.C.-C.); UO230010@uniovi.es (F.-A.-N.); vanesa.cepas@gmail.com (V.C.); sheilafdzvega@gmail.com (S.F.-V.); iquirosg@gmail.com (I.Q.-G.); sainzrosa@uniovi.es (R.M.S.); 2 Instituto de Investigación Sanitaria del Principado de Asturias (ISPA), Hospital Universitario Central de Asturias (HUCA), Avda. Roma s/n, 33011 Oviedo, Spain; 3 Institut de Genetique Moleculaire de Montpellier, University of Montpellier, CNRS, 34095 Montpellier, France; pedrogonzalezmenendez88@gmail.com

**Keywords:** melatonin, melanoma, B16-F10, cytoskeleton, metastasis

## Abstract

Neuroindole melatonin, a hormone synthesized during the night mainly—but not exclusively—by the pineal gland of all vertebrates, functions as an adapting signal to the light-dark cycle. Its antioxidant, neuroprotective, anti-inflammatory, and antitumor properties are all well-known and widely reported. Melanoma is one of the most common carcinomas among developed countries and a type of tumor particularly difficult to fight back in medium/advanced stages. In contrast to other types of cancer, influence of melatonin on melanoma has been scarcely investigated. Thus, we have chosen the murine melanoma model B16-F10 cell line to study antiproliferative and antitumoral actions of melatonin. For this purpose, we combined both, cell culture and in vivo models. Melatonin reduced either, growth rate or migration of B16-F10 cells. Furthermore, melanin synthesis was altered by melatonin, promoting its synthesis. Melatonin also induced a G2/M cell cycle arrest and altered the cytoskeletal organization. To corroborate these results, we tested the effect of melatonin in the in vivo model of B16-F10 cell injection in the tail vein, which causes numerous lung metastases. Two different strategies of melatonin administration were used, namely, in drinking water, or daily intraperitoneal injection. However, contrary to what occurred in cell culture, no differences were observed between control and melatonin treated groups. Results obtained led us to conclude that melatonin exerts an antiproliferative and anti-migrating effect on this melanoma model by interfering with the cytoskeleton organization, but this pharmacological effect cannot be translated in vivo as the indole did not prevent metastasis in the murine model, suggesting that further insights into the effects of the indole in melanoma cells should be approached to understand this apparent paradox.

## 1. Introduction

The pineal indole melatonin is a tryptophan-derived hormone, synthesized and released by the pineal gland during the dark period in all vertebrates [1]. Even though this gland is the organ responsible for the serum concentrations at night, both precursors and synthetic enzymes as well as melatonin itself have been found in many other tissues including the Harderian gland, retina, and gut among others [2,3,4]. However, the contribution to the systemic circulation by these tissues is minimal—if any—as it is deduced from the fact that pinealectomy leads to a total drop in melatonin serum levels and, as a consequence, the lack of circadian rhythmicity [5]. Melatonin has been highly conserved during evolution which has recently led to propose the well-known antioxidant activity of the indole as the first function, since there is evidence of its presence in primitive organisms with aerobic metabolism. Therefore, melatonin would help in eliminating reactive oxygen species generated during oxidative phosphorylation [6]. In vertebrates, however, melatonin displays different functions, the most important being the biological clock indicating the light-dark period, and regulating the reproductive cycle, thus fulfilling an important role in organisms with seasonal reproduction [7,8].

At cellular level, melatonin can exert its functions in different ways. Firstly, by binding to specific G-coupled membrane receptors (i.e., MTNR1A, MTNR1B, and MTNR1C), which possess potential glycosylation and phosphorylation sites for various proteins such as protein kinases A and C (PKA, PKC). The corresponding associated G proteins will in turn give rise to signaling cascades that ultimately end up modifying different intracellular routes [9]. As an alternative, a receptor-independent, direct antioxidant ability associate with its chemical structure has also been well documented. In this way melatonin molecule can donate one or more electrons to free radicals, thus neutralizing its oxidizing activity or can act synergistically with other antioxidant molecules such as ascorbic acid or α-tocopherol [10]. 

In addition to its broad physiological properties, numerous research groups have reported different antitumor effects of the indole on several tumor types including breast, colon, ovary, endometrium, prostate, liver and skin cancer cells, among others [11,12,13,14,15,16,17]. Furthermore, the indole is capable of inhibiting cell growth, either leading tumor cells to a cell cycle blockage at some stage, acting as an antagonist of other pro-tumorigenic molecules such as estradiol in breast cancer, or by preventing the activation of the androgen receptor in prostate cancer [18,19,20]. Another reported antitumor activity of melatonin relates to its ability to regulate the cell death processes. Although the role of melatonin as an apoptosis inhibitor in immune and neuronal cells was described more than two decades ago, more recently some authors have also shown that melatonin may have proapoptotic effects on tumor cells, commonly associated to its ability to inactivate NF-κB or to increase specific cytokine levels such as IL-6 [21].

The skin is one of the major defensive barriers against the environment, and also an organ where melatonin synthesis has been demonstrated [22]. Skin has all the molecular and biochemical machinery to produce melatonin and is mainly located in the upper layers of the epidermis [23]. Therefore, one of its main functions is the protection against UV radiation which, due to its high energy, can cause harmful alterations at genetic and cellular level, being therefore one of the main etiological agents involved in the development of skin cancer [24]. Among skin tumors, melanoma is frequently characterized by its poor prognosis and low survival rate [25]. These pigmented tumors are highly invasive and metastatic, generally appearing in cutaneous zones but also other tissues, i.e., intestine, oral cavity, or eye among others [26]. According to WHO, there are roughly 132,000 new cases of melanoma each year worldwide, of which 66,000 are fatal [27]. In addition to other well-demonstrated functions including the reduction of cell proliferation [28] or induction of cell differentiation [29], melatonin is involved in the modulation of melanin synthesis pathway. An increase in melanin pigment levels after melatonin treatment in vitro has been described [30]. In addition, due to the high incidence and severity, it is necessary to search for new molecular routes and drugs that will reduce or halt the development and progression of melanoma. Melatonin, as already described above, has different antitumor properties and specifically in melanoma cells, several groups have previously shown that treatment with melatonin triggers a suppressive effect of cell growth mediated by an increase in apoptosis levels in melanoma cell lines SBCE2, WM-98, WM-169, or SKMEL-188 [9], mainly throughout the interaction with specific membrane receptors. Other laboratories have described that melatonin can induce apoptosis in the murine B16-F10 melanoma model but when combined with other death-inducing agents such as endoplasmic reticulum, stress-inducing drugs [28].

Since melanoma is considered one of the tumors with worse prognosis in Western countries and given the important antitumor and antioxidant activities of melatonin which make it an interesting molecule for the study of cancer therapies, the main objective of this work is to validate the antitumor role of the neuroindole pineal melatonin both in vitro and in vivo in the murine melanoma cell model B16-F10.

## 2. Results

### 2.1. Antiproliferative Effects of Melatonin In Vitro

**Figure 1:**
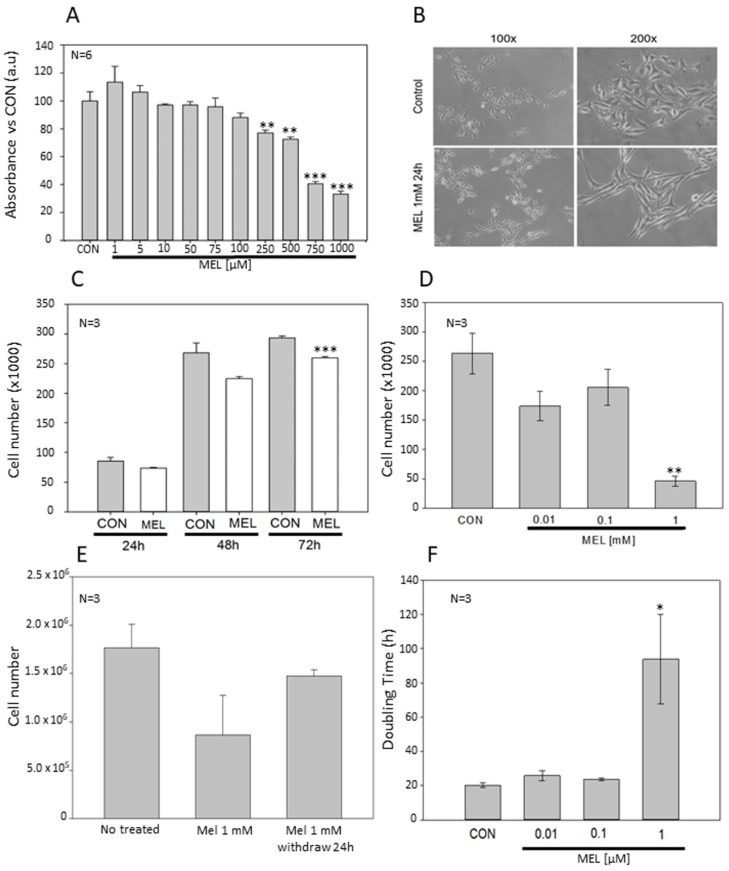
Antiproliferative effect of Melatonin in B16-F10 melanoma cells. (**A**) MTT cell viability assay. Cells were seeded in 96 Wells plate at the indicated concentrations, after 72 h MTT reduction levels were assessed. (**B**) B16-F10 micrographs from control or melatonin-incubated cells were taken at a final magnification of 100× and 200×. (**C**) Average cell number after 24, 48, and 72 h of treatment with 0.1 mM of melatonin. (**D**) Average cell number after 72 h of treatment with different concentrations of melatonin. (**E**) Average cell number after 24 h of treatment with 1mM of Melatonin and withdraw treatment other 24 h. (**F**) Cell culture doubling time of every experimental group after treatment with melatonin during 72 h. Data were shown as average +/− SEM. Significance vs. CON. * *p* < 0.05, ** *p* < 0.01, *** *p* < 0.001.

**Figure 2:**
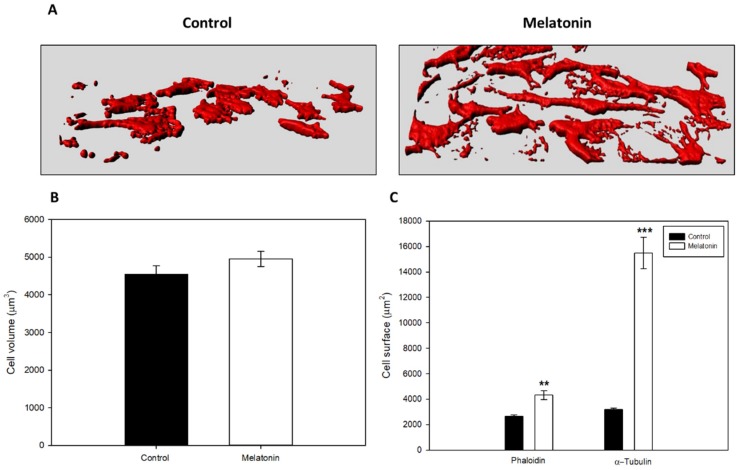
Morphological changes of B16-F10 cells after 24 h of treatment with melatonin. (**A**) 3D reconstruction of cell culture based on F-Actin distribution. Red areas represent the surface occupied by F-Actin (**B**) Average cell volume based on F-Actin distribution. (**C**) Average cell surface based on F-Actin distribution and α-tubulin. Data were shown as average +/− SEM. Significance vs. CON. ** *p* < 0.01, *** *p* < 0.001.

Since melatonin displays antiproliferative effects on tumor cells, including different types of skin cancer cells, we assayed whether viability, cell growth, and doubling time of B16-F10 cells change after incubation with the indole. By using 3-(4,5-dimethylthiazol-2-yl)-2,5-diphenyltetrazolium bromide (MTT) reduction to formazan as cell viability/proliferation assay, here we show that melatonin induces a decrease in MTT reduction in a dose-dependent manner, and this reduction was significant at concentrations above 250 μM (Figure 1A). Since these data could indicate an increase in cell death, or a decrease in cell growth, morphology was checked using phase-contrast microscopy and no symptoms of cytotoxicity were observed, so we hypothesized that melatonin produced a decrease in growth rate without affecting cell death in our in vitro melanoma model (Figure 1B). To corroborate this result, we analyzed how both cell number and doubling time changes after treatment with melatonin. Cell counting showed a significant reduction in the cell number in a concentration-dependent manner, obtaining statistically differences with concentrations of 1 mM melatonin after 72 h treatment (Figure 1C). Furthermore, it was also studied how different concentrations of melatonin regulated cell growth, obtaining a significant reduction with 1 mM melatonin, as in the previous experiment (Figure 1D). To test whether the presence of the indole was required permanently in the cell culture medium for its action, we changed melatonin-containing medium by regular culture medium and let cells grow again. Cells partially recovered the growth rate after the removal of the indole, even though number of cells did not reach the levels of the control group (Figure 1E). After 72 h of growth, doubling time in melatonin-treated cells (1 mM) significantly increased respect to the control group (Figure 1F). However, some morphological changes were observed after incubation with the indole. Thus, melatonin induced an elongated, fibroblast-like morphology in melanoma cells, when compared to the common rounded and spindle-shaped morphology observed in control cells. For a quantitative study, two morphometric parameters such as volume and cell surface were analyzed. Three-dimensional reconstruction of cultured cells did not show significant differences between volumes in both groups (Figure 2A,B). However, specific staining of either actin microfilaments or microtubules showed that cells treated with melatonin occupied a larger surface area than the control cells (Figure 2C).

### 2.2. Melatonin Detection in Cell Culture by High Performance Liquid Chromatography (HPLC)

Extraction and quantification of melatonin were performed and assayed in both, extracellular culture medium and intracellular content of B16-F10 cells. The internal standard previously added (5-methoxy-tryptophol) displayed a 6.35 min retention peak. Samples from melatonin-incubated cells showed a characteristic peak at a retention time of 7.39 min, with a maximum absorption spectrum at 279 nm wavelength, both corresponding to the retention time and absorption spectra of melatonin, identical to that of the melatonin standard used. No peak was observed in control groups (Figure S1A,B). A total of 15.35 pmol/10^6^ cells were detected within the B16-F10 cells after 72h of melatonin culture. Culture media from these indole-treated cells showed a total concentration of 0.88 mM after 72 h of culture, indicated a relatively low uptake of melatonin by these cells. 

### 2.3. G2/M Cell Cycle Arrest Induced by Melatonin Treatment

**Figure 3:**
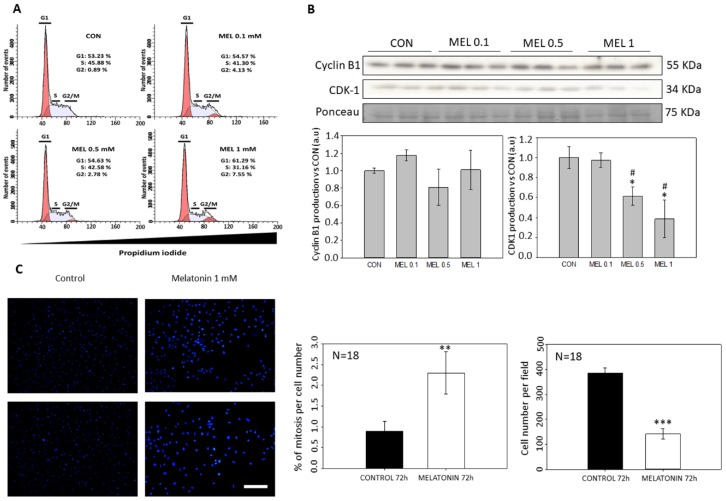
Cell cycle arrest induced by melatonin. (**A**) B16-F10 cells distribution around cell cycle after treatment with different concentrations of melatonin. (**B**) Analysis of proteins involved in G2/M checkpoint. (**C**) Percentage of mitosis per cell number in control and treatment group. Blue dots represent nuclear staining of DNA with DAPI. Significance vs. CON. * *p* < 0.05, ** *p* < 0.01, *** *p* < 0.001. Significance vs. MEL 0.1. # *p* < 0.05. Image Scale bar = 100 µm.

**Figure 4:**
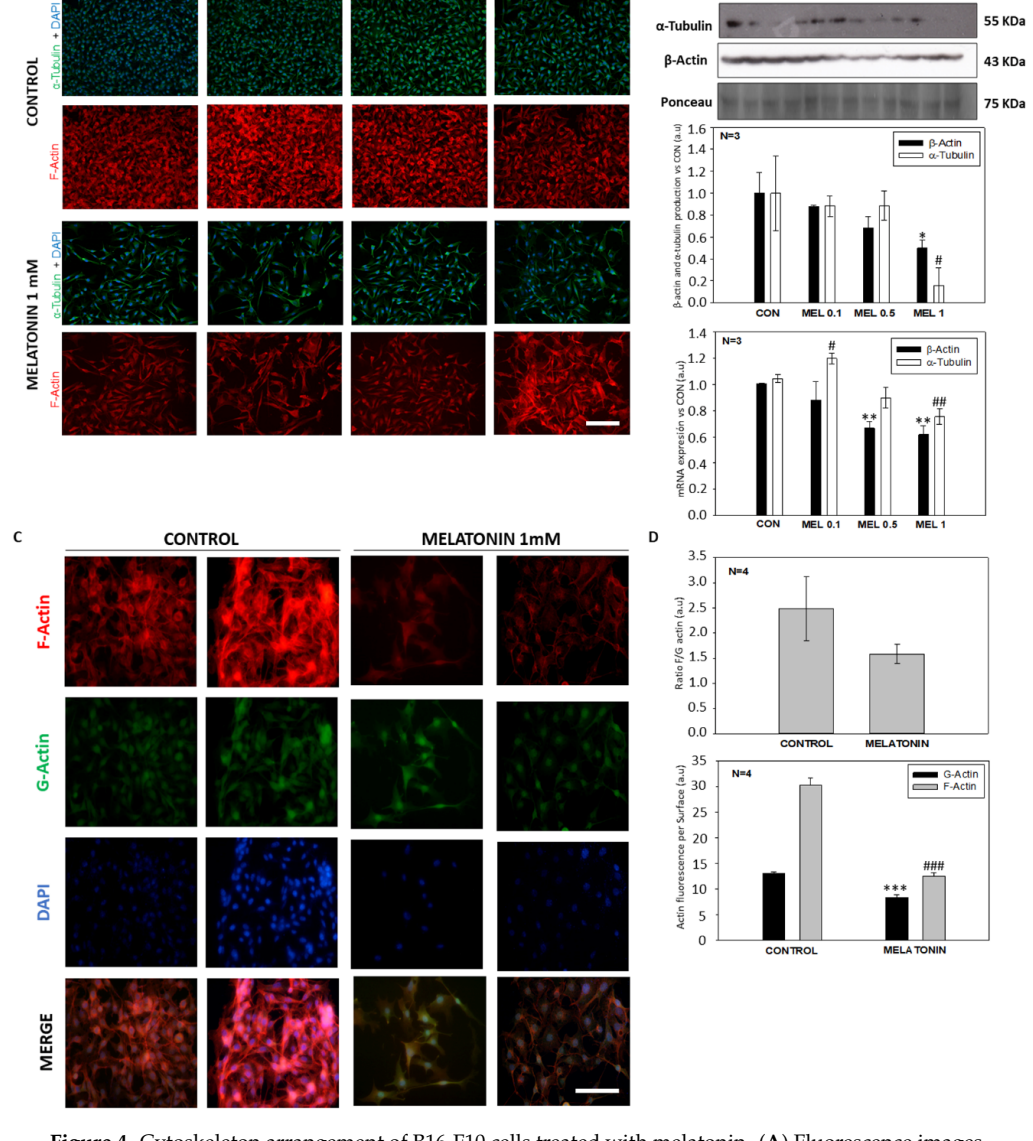
Cytoskeleton arrangement of B16-F10 cells treated with melatonin. (**A**) Fluorescence images of α-tubulin and β-actin location in control and treated cells. (**B**) Western blot and qPCR analysis of protein and mRNA levels of α-tubulin and β-actin. (**C**) Fluorescence images of G and F actin in control and treated cells. (**D**) Quantification of ratio between F and G actin and fluorescence level of both proteins. Significance vs. β-Actin CON. * *p* < 0.05, ** *p* < 0.01. Significance vs. α-Tubulin CON. # *p* < 0.05, ## *p* < 0.01. Significance vs. G-Actin CON. *** *p* < 0.001. Significance vs. F-Actin CON. ### *p* < 0.001. Image Scale bar = 100 µm.

Since melatonin decreased mitochondrial MTT reduction due to a decrease in the growth rate without increasing cell death, the specific effect of the indole on the cell cycle distribution was studied. To this aim, cells were analyzed by flow cytometry after 24 h of incubation with the indole. The study revealed an increase in the number of cells present in G1 and G2/M phases on detriment of the S phase in the groups treated with melatonin, thus indicating a G2/M arrest (Figure 3A). To study whether there was a halt in the cell cycle, analysis of the main proteins involved in these checkpoints was performed by Western blot. While no alteration in Cyclin B1 levels was observed, CDK1 levels were significantly reduced in melatonin-treated cells (0.5 and 1 mM) compared to control cells, which might account for an arrest in G2/M phase (Figure 3B). Furthermore, the total number of mitosis in melatonin-treated cells doubled those found in control groups (Figure 3C). These results prompted us to study the possible reorganization of the cytoskeleton components, as they play an important role in the progression of mitosis and cytokinesis and have important effects on cell morphology. When quantifying both, β-actin and α-tubulin, a decrease in the fluorescence intensity of both proteins was observed in the treated groups respect to the controls (Figure 4A). Furthermore, these results were corroborated by the levels of total protein production as well as the total mRNA expression (Figure 4B). The analysis of G:F actin ratios showed no differences between control and treated cells. Nevertheless, control cells exhibited a higher fluorescence of both proteins, when compared to melatonin-incubated cells (Figure 4C,D).

### 2.4. Melatonin Slows down the Wound-Healing

**Figure 5:**
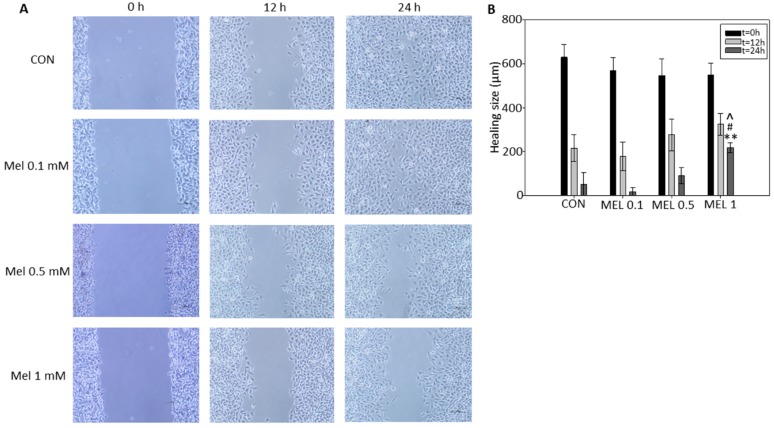
B16-F10 cell migration after incubation with different concentrations of melatonin. (**A**) Wound healing assay of each experimental group, images were taken at a magnification of 40. (**B**) Wound healing average size of each experimental group. Data were shown as average +/− SEM. Significance vs. CON 24h. ^ *p* < 0.05, significance vs. Mel 0.5 24h. # *p* < 0.05, significance vs. Mel 0.1 24 h. ** *p* < 0.01.

Given that melanomas are highly invasive and metastatic tumors, migration of the B16-F10 cells by wound healing assay was studied. After incubation with melatonin at the concentrations previously indicated, images were taken at the established time courses. As can be observed (Figure 5), wound closing speed was significantly lower in melatonin treated cells compared to control group. After 24 h wound had almost disappeared. Additionally, the rate of wound closure was dependent in a concentration-manner, since the size of the wound in the culture is significantly greater at 24 h in the group treated with 1 mM melatonin than in the rest of the melatonin-treated groups (Figure 5).

### 2.5. Intracellular Melanin Synthesis Induced by Melatonin

**Figure 6:**
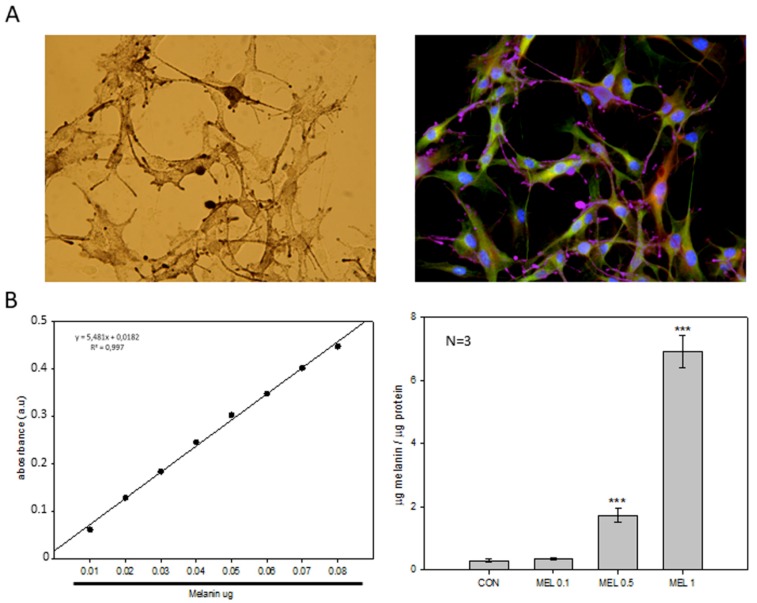
Melanin localization and quantification in B16-F10 cells. (**A**) Phase-contrast micrographs of intracellular melanin depots (left) and the corresponding merge with α-tubulin and β-actin distribution (right). Micrographs were both taken at a final magnification of 200×. (**B**) Quantification of total intracellular melanin after treatment with different concentrations of Melatonin. Data were shown as average +/− SEM. Significance vs. CON. *** *p* < 0.001

When cells were observed under phase-contrast microscopy, cells treated with melatonin appeared with dark pigmentation in the cytoplasm, mainly located in the periphery, which seemed to correspond to melanin, the characteristic pigment found in melanocytes and melanoma cells (Figure 6A). To quantify the number of pigmented cells in control and melatonin treated groups, the total content of intracellular melanin was analyzed. Previously, for this purpose, optimal melanin extraction time was estimated at 60 min, using synthetic melanin as standard. A calibration curve of extracted melanin from the B16-F10 cells was obtained. Incubation with 100 μM, 500 μM, and 1 mM melatonin for 24 h induced an increase in intracellular melanin levels in a dose-dependent manner, statistically significant when concentrations were over 100 µM (Figure 6B).

### 2.6. Study of the Levels of Antioxidant Enzymes Involved in the Synthesis and Scavenging of Hydrogen Peroxide

**Figure 7:**
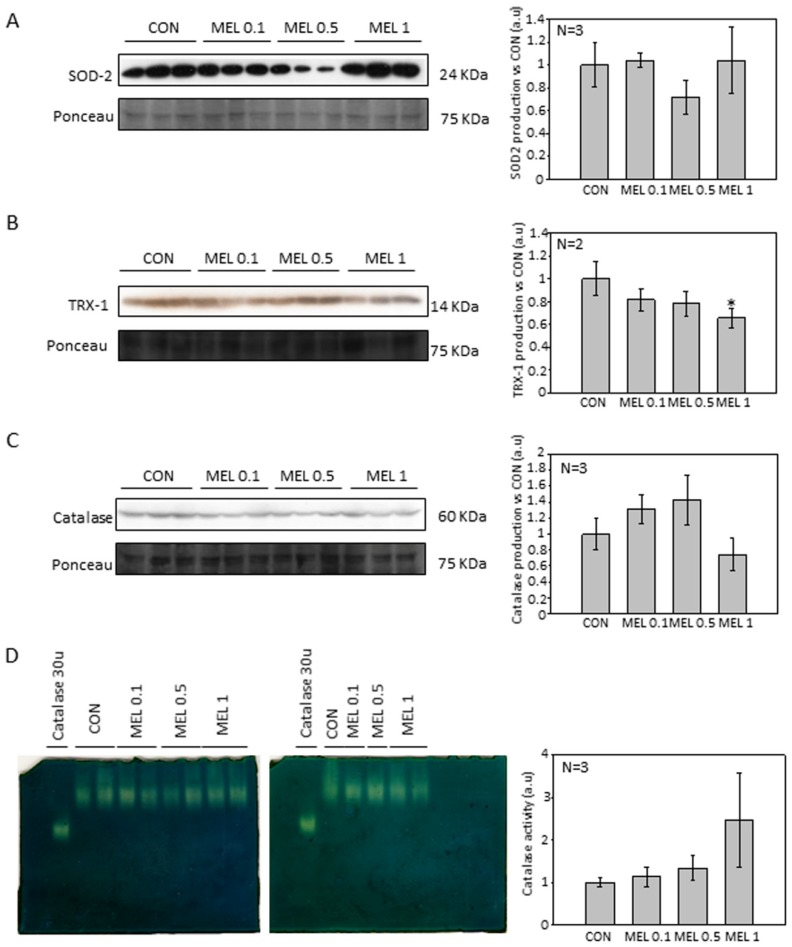
Study of redox proteins included in hydrogen peroxide production and scavenging after melatonin treatment. (**A**) SOD2 total protein production. (**B**) TRX-1 total protein production. (**C**) Catalase total protein production (**D**) Catalase activity gel. Data were shown as average +/− SEM. Significance vs. CON. * *p* < 0.05.

It has been previously demonstrated that melatonin inhibits cell growth by either, directly decreasing ROS levels, or by increasing the expression and production of antioxidant enzymes. Besides, it has been described that hydrogen peroxide is one of the most important free radicals involved during melanin synthesis. Knowing the role played by reactive oxygen species such as hydrogen peroxide in the melanin synthesis process, the total amount of antioxidant enzymes SOD2, CAT and TRX1 present were assayed in control or melatonin-incubated cells, since those are the enzymes responsible for ROS scavenging. No significant differences in the total amount of SOD2 or CAT protein levels were found (Figure 7A,B). However, TRX-1 levels decreased after treatment in a dose-dependent manner, reaching statistical significance in the groups treated with 1 mM melatonin (Figure 7C). On the other hand, we also studied the change in the enzymatic activity of CAT, because it is a major enzyme implicated on hydrogen peroxide scavenging. Despite the fact that there was no difference in protein production, we observed an increase in CAT activity in the extracts of melatonin-treated cells although it was not significant (Figure 7D).

### 2.7. Melatonin Effects on Melanoma Mice Model In Vivo

**Figure 8:**
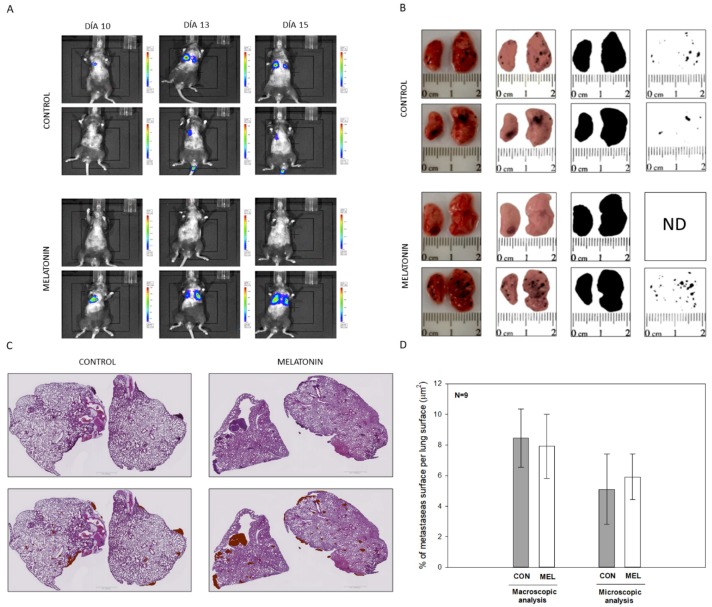
In vivo model for B16-10 lung metastasis treated with IP melatonin. (**A**) Bioluminiscence localization and size of total lung metastases. (**B**) Macroscopic images of lung metastases and image isolation process. (**C**) Microscopic scanning of lung sections and detection of metastatic tissue. (**D**) Percentage of metastases Surface per total lung surface. Data were shown as average +/− SEM. ND: not detectable.

Finally, to corroborate our in vitro results, a murine model was used to study the in vivo effects that melatonin on cell metastasis after injecting the cells into the tail vein of C57BL/6J mice. As a first approach, imaging techniques to locate how cells nest and proliferation in the lung evolved were used. IP injection of luciferin-containing cells in mice indicated that bioluminescence could be observed 10 days after injection. In most animals, migration occurred first in the right lung, and from day 13 and on both lungs were found affected (Figure 8A). After the animals were sacrificed, macroscopic quantification of the metastases surface and their ratio respect to the total lung area was recorded, using a software macro specially designed for image J. No significant differences between melatonin-treated animals and controls were detected (Figure 8B,D). Likewise, and as a reinforce for this study, microscopic analysis of lung sections of each group revealed again no differences. Microscopical observation showed that most of metastases were located in the peripheral area of the lung in both experimental groups, and in some cases, mice displayed metastases variable in size in more internal areas of the tissue. As in the case of the microscopy quantifications, no differences between the area occupied by metastasis in the lungs treated with IP injection of melatonin compared to the controls treated with vehicle were observed (Figure 8C,D).

Since we did not find differences in the migration of B16-F10 cells after the IP injection of melatonin, a new experiment with a different administration method was performed, in this case by adding melatonin in the drinking water. Similarly, as in the previous experiment, we also monitored the image by bioluminescence, corroborating that 10 days after the injection of the cells in the tail vein, most of the animals still did not show visible metastases in the lung, whereas after 15 days both lungs were already affected (Figure S2A). Again, the microscopical study of the metastatic surface respect to the total lung surface analyzed revealed no statistical differences between vehicle-treated or melatonin-treated groups (Figure S2B,C). Furthermore, there not exists differences in water intake between control and treatment animals over the experiment (Figure S2D).

## 3. Discussion

As described earlier, the pineal neuroindole melatonin has important antitumor actions, among which the antiproliferative effect stands out [31]. Previous studies have shown that concentrations ranging from high micromolar to 1 mM of this indole reduce cell proliferation and increase levels of cell death in this cell type [24]. However, it has not only been described in this cell type but also been found to perform the same effect on other tumor types of melanoma, as well as in breast cancer and prostate cancer among others [21,32].

Results show that increasing concentrations of melatonin in the high micromolar range have a direct impact on cell proliferation, following a dose-dependent decreasing trend. In addition, the presence of indole is necessary for its effects to be maintained, since once removed, cell proliferation immediately recovers to a normal growth rate. Contrary to other studies using different cell lines, no signs of cytotoxicity and cell death were observed. Kim and co-workers have reported that melatonin compromises cell survival using the same cell model, i.e., B16-F10 cells. However, in this study, they combined the indole with the induction of endoplasmic reticulum stress, and they observed that melatonin enhanced p-PERK. Furthermore, cell death is significantly increased when the indole is combined, in a dose-dependent manner [28]. Microscopically, we did not detect any morphological signs of damage as compared to the control group. However, morphologically, a larger, in relation to this section we observed that, although they did not show differences in cell volume, they occupied a greater surface area in the culture plate, thus as they were elongated, acquiring a morphology like fibroblasts. This is due to a reorganization of the cell cytoskeleton mediated by the action of melatonin, which also has different effects on the physiology of the cell altering other processes such as mitosis [33]. Furthermore, melatonin induces apoptosis in DLD1 ovarian colon cancer and A2780 ovarian cancer cells line after 24 h of treatments [34]. In human SK-LU-1 lung cancer cells, an increase in melatonin concentration was correlated with the increase in the concentration of apoptotic bodies [35].

By not observing signs of cytotoxicity in the cells, we studied whether melatonin exerted that antiproliferative effect in vitro due to an arrest at some stage of the cell cycle, since as we have mentioned previously other groups have demonstrated the effect of melatonin as an antiproliferative molecule when blocks the cells in the G0 and G1 phases of the cell cycle [36,37]. When analyzing the cell cycle in B16-F10 cells, we observed that the groups treated with melatonin had a greater number of events in G1, as previously published by other groups, as well as in G2/M. When studying proteins involved in the passage of the G2 phase cells to mitosis, we observed how there were alterations in CDK-1 levels, which occurred in smaller amounts in those groups treated with melatonin, which would prevent cells from entering mitosis at the same rate as the control which explains the reduction in cell number increment under melatonin treatment. In addition to this, we observed a greater number of mitoses in the groups treated with melatonin. This phenomenon could be related to the stop in the cell cycle, since the melatonin retaining the cells in G2/M for a longer period would cause this increase in the number of mitosis. On the other hand, and in relation to this phenomenon, we study the state of the cellular cytoskeleton, since as previously described by other groups, melatonin acts as a modulator of cytoskeleton proteins, altering the actin microfilaments to a greater extent, although It also acts at the level of microtubules, which are highly related to the mitotic processes of anaphase, telophase and cytokinesis [38,39]. When the levels of these proteins are altered, mitosis can suffer abnormalities that lead to the increment of the growth delay, as in our case.

In order to end cell proliferation studies in this model, we finally studied the effect that melatonin has on cell migration phenomena, since it is described both in vitro and in vivo in other models that this indole is a potent antiproliferative, inhibiting the growth and migration of tumor cells. It has been previously described by other research groups that melatonin reduces the migration rate by wound healing test, either by administering it individually or as an adjuvant with other treatments, in different tumor models such as squamous carcinomas of esophagus, gliomas, or melanoma itself, thus coinciding with our results, where we see how melatonin reduces the speed of wound closure in treated cells with respect to control cells [40,41].

An important feature of melanocytes is their ability to synthesize the melanin intracellular pigment. Melatonin is a molecule capable of causing the aggregation of melanosomes in those cells that present these organelles, and since the B16-F10 cell model is capable of synthesizing this pigment we decided to study the phenomenon, observing how the administration of melatonin to cells caused an increase in the synthesis of the melanin pigment, which was mainly distributed in the cell periphery, near the plasma membrane. As described above, melanin has the main function of protecting the skin from ultraviolet radiation, which can trigger mutagenic phenomena in the DNA which can trigger the appearance of tumorigenic processes [42]. Since during the synthesis of melanin oxidation reactions are triggered reduction where superoxide anion free radicals (O_2_^−^.) And hydrogen peroxide (H_2_O_2_) occur, mainly by the key enzyme of the synthesis pathway, tyrosinase, which It transforms the amino acid tyrosine to 3,4-Dihydroxyphenylalanine and later to dopaquinone, and due to the pro-tumorigenic effect that these free radicals present in tumors, we decided to analyze how levels of both production and activity of different antioxidant proteins varied [42,43]. In addition, different research groups have studied the levels of antioxidant proteins, mainly involved in the production and purification of O_2_^−^. in this cellular model, observing how melatonin treatment reduced the levels of both SOD2 and catalase [28]. When analyzing our results, we observed that the levels of the different antioxidant proteins studied were not altered, with the exception of thioredoxin 1 (TRX-1), which significantly decreased their production when cells were treated with melatonin, in relation to catalase, we observed also a decrease in production, but its levels do not reach statistical significance. Therefore, our results agree to some extent with those already published. However, we do not observe large differences in our model.

Altogether this result allows us to conclude that melatonin caused a reduction in cell proliferation in the melanoma cell model B16-F10. Then we expand our study towards an in vivo model of lung metastases in order to check if, at the systemic level, melatonin caused a decrease in the ability to migrate and metastasize in B16-F10 cells, which has not been previously described. To do this, we carried out two approaches as we have already described, administrating melatonin IP and using drinking water. In addition, the beneficial effects of the administration of melatonin in experimental mice in relation to other tumor models such as prostate cancer have been previously described by our group and others [43,44]. When analyzing our results, we did not observe differences in the number of metastases or at the macroscopic or microscopic level, which makes us think that due to the aggressiveness of these cells during the metastatic process, which already affects both lungs after 15 days of injection of the cells in the tail of the mouse, melatonin is not able to alter the rate of proliferation and migration of the cells. In contrast to in vitro model, where melatonin was directly applied within the culture medium, its availability in the lungs of our in vivo model might be compromised.

## 4. Materials and Methods 

### 4.1. Cell Culture

Murine melanoma tumor cell line B16-F10 (ATCC^®^, CRL-6475™, Manassas, VA, USA,) was employed for the studies. This cell line was originated from the parental line B16 and it is characterized by its ability to form colonies (nodules) in the lung after intravenous injection in in vivo models [29]. Unless otherwise indicated, all reagents were purchased from Sigma (Sigma-Aldrich Inc., St Louis, MO, USA). Cells were cultured in Dulbecco’s Modified Eagle’s Medium (DMEM) (Sigma) supplemented with 10% fetal bovine serum (FBS) (Sigma), 10mM HEPES, 2mM L-glutamine (Lonza, Basel, Switzerland) and 1% antibiotics and antifungals (amphotericin B, penicillin and streptomycin) (Gibco, Grand Island, NY, USA). Cells were kept under controlled conditions in a CO_2_ incubator (New Brunswick ™ Galaxy^®^170s, Eppendorf, Germany) at 37 °C and 5% CO_2_ atmosphere. Before carrying out each experiment, at least 24 h were expected from the time of seeding to favor the adhesion of the culture to the substrate.

### 4.2. MTT Cell Viability Assay

Cells were seeded in a 96-well plate at a density of 1500 cells/well in 100 μL of complete culture medium and left attach overnight. After 24 h, melatonin (ranging from 1 μM to 1 mM) was added (Merck, Kenilworth, NJ, USA). The indole was dissolved at 1M stock solution in dimethyl sulfoxide (DMSO) (Sigma) and the corresponding DMSO concentration was added to control cells. After 72 h of incubation, MTT reagent was added at a final concentration of 0.5 mg/mL and allowed to react for 4 h. Then, one volume of lysis buffer solution (20% SDS in 50% dimethylformamide, pH = 4.7) was added and incubated in the dark at 37 °C overnight. The absorbance was measured at a spectrophotometer microplate reader Cary 50 MPR (Varian, Palo Alto, CA, USA) at a wavelength of 570 nm using absorbance at 690 nm as reference wavelength.

### 4.3. Proliferation and Doubling Time Assay

To estimate both, cell proliferation and the doubling time, i.e., time required to double their number, a duplication test was designed. Cells were seeded in 6-well plates at a density of 12,500 cells/mL. Three replicates of each experimental group were seeded in the presence of 10 μM, 100 μM or 1 mM melatonin. After 72 h, cells were collected by scraping with a rubber policeman and fixed in 500 μL of 70 % ethanol at 4 °C. Cells were then counted using a hemocytometer. The doubling time was calculated based on the following mathematical formula.

d = t × log(2)/log(Cf) − log(Co)(1)

### 4.4. Quantification of Intracellular and Extracellular Melatonin in Cell Culture

To quantify the total content of melatonin present in the culture medium, and the intracellular content, a high-performance liquid chromatography (HPLC) assay was performed. To this aim, cells were seeded at the density of 2.5 × 10^4^ cell/mL in 100 mm plates. After 24 h, melatonin was added at a concentration of 1mM. After 48 h, cells and their respective culture medium were collected, and an organic extraction was carried out based on a protocol previously described by our research group [31]. As a reference, melatonin concentration was calculated using a melatonin calibration curved performed from 0.1 ppm to 10 ppm, as well as an internal standard to check the quality of the extraction. 

### 4.5. Cell Cycle Analysis

For these experiments, *c*ells were seeded in 100 mm diameter plates at a density of 4 × 10^4^ cells/mL and left to reach 75–80% confluence. Melatonin was added at concentrations of 100 μM, 500 μM, and 1 mM, and after 24 h cell cycle was analyzed. For this purpose, cells were harvested by trypsinization and centrifuged at 500× *g* for 5 min at 4 °C. After removing supernatant, cells were washed twice with ice-cold PBS, then fixed in 500 μL of 70% ethanol and stored at 4 °C until analysis. At the time of analysis, cells were centrifuged for 5 min at 700× *g*, and cell pellet was resuspended in 500 μL of a staining solution (propidium iodide 100 μg/mL, RNase 100 U/mL, 10 mL PBS with 1 g/L of glucose). Finally, it was incubated at RT for 30 min and the samples were analyzed on a Cytomics FC500 flow cytometer (Beckman Coulter, Indianapolis, IN, USA).

### 4.6. Wound Healing Assay

To this purpose, cells were seeded in 6-well plates and left to reach confluence. Then, scratches wounds were made by scraping the cell layer across each well using a 100 μL pipette tip. Then after washing with PBS to remove the detached cells, melatonin was added at 100 μM, 500 μM, and 1 mM in fresh complete culture medium. Micrographs of the scratched area were taken at the beginning (t = 0) and at 12 and 24 h, using the 40× magnification objective.

### 4.7. Extraction and Quantification of Intracellular Melanin

Cells were seeded at concentration of 2.5 × 10^4^ cell/mL in 100 mm plates. When cells reached confluency, melatonin was added at the indicated concentrations. After 24 h, cells were collected by scraping in 1 mL of PBS and centrifuged for 5 min at 500× *g*. Pellets were resuspended in 500 μL of 1 × PBS and sonicated in an ultrasonic bath. Afterward, samples were centrifuged at 12,000× *g* for 20 min at 4 °C and the supernatant was collected in a clean tube for protein precipitation by 10% trichloroacetic acid, and then quantified by the Bradford method (Sigma) [45].The pellet was dissolved in 1 mL of NaOH 1N and 10% DMSO and heated in a heater at 80 °C for 1 h. After the incubation the absorbance was quantified at 470 nm. The melanin concentration was calculated using a synthetic melanin standard (Sigma) at concentrations from 0.01 to 0.1 mg of melanin.

### 4.8. SDS-PAGE and Immunoblotting

Cells were seeded in 100 mm plates at 2.5 × 10^4^ cell/mL and left to reach 80% confluency. Then melatonin (ranging from 100 μM to 1 mM) was added. For protein extraction, cells were scraped with a rubber policeman in culture medium and subsequently centrifuged at 500× *g* for 5 min. After medium removal, RIPA lysis buffer (0.1% sodium dodecyl sulphate (SDS), Igepal C 1%, 0.5% Sodium Deoxycholate, with 150 mM sodium chloride (NaCl) in a 50 mM solution of Tris-HCl at pH 7.4) with freshly added protease inhibitors (2 μg/mL Apoprotein A, 10 μg/mL Leupeptin, 1 μg/mL pepstatin, 200 μM sodium orthovanadate, 1 mM sodium fluoride (NaF), 1 mM phenylmethylsulphonyl fluoride (PMSF) and 1 mM dithiothreitol (DTT) was added. Samples were incubated on ice for 30 min and subsequently centrifuged at 15,000× *g* for 15 min at 4 °C. 

Proteins (50 µg) were resolved in 12% acrylamide/bisacrylamide (Bio-Rad, Hercules, CA, USA) gels with 15-lane combs and 1.5 mm spacer crystals in which 50 μg of protein from each sample was loaded with 4× loading buffer (250 mM Tris-HCl pH 6.8, 40% glycerol, 8% SDS, 0.2% bromophenol blue, 28 μL/mL β-mercaptoethanol). Electrophoresis and transfer were performed in a Miniprotean3 (Bio-Rad, Hercules, CA, USA). Once the electrophoresis was performed, proteins were transferred to PVDF immobilon-P membranes (Millipore, Burlington, MA, USA). Subsequently, membranes were stained with a solution containing 0.1% (w/v) Ponceau in 5% acetic acid (v/v) (Bioquochem, Siero, Asturias, Spain) and blocked for one hour in a 5% solution of non-fat milk powder (BD, Sparks, MD, USA) in TBS-T (Tris-HCl, 20 mM, 137 mM NaCl, and 0.05% Tween-20 pH 7.4). After blocking the membranes were incubated with the primary antibodies at 4 °C overnight using the concentrations indicated in the following table (Table 1).

### 4.9. Catalase Activity Native Gel

Cells were seeded in 100 mm plates until they reached 75–80% confluence. Then, melatonin at 0.1 mM, 0.5 mM, or 1 mM was added. After 24 h, cells were harvested by trypsinization, centrifuged for 5 min at 500× *g*, resuspended in PBS supplemented with 0.5% IGEPAL C and incubated on ice for 30 min. After centrifuging lysate for 15 min at 15,000× *g*, supernatant was transferred to another tube and protein quantification was performed using the Bradford method. After electrophoresis gels were washed in milli-Q water to remove the remaining buffer and incubated for 10 min in 0.003% hydrogen peroxide (Sigma). Hydrogen peroxide was then removed and two staining solutions containing 2% ferric chloride and, 2% potassium ferrocyanide were added until white bands shoring the catalase activity were displayed at in the expected native protein weight (i.e., 233 kD). Reaction was stopped by removing the stain and washing with mili-Q water. Gels were scanned, and band intensity was quantitated by densitometry, using the ImageJ software (version 1.49P, NIH, Bethesda, MD, USA) downloaded from the following link https://imagej.nih.gov/ij/index.html. 

### 4.10. RNA Isolation and qPCR Analysis

For RNA isolation, cells pellet was homogenized and extracted using Tri-Reagent procedure (Sigma) according to the manufacture protocol, next we quantized the total concentration and analyzed their stability in agarose gel. cDNA was synthetized from 1 µg of RNA extraction using NG dART RT-PCR kit (EURx, Gdansk, Przyrodników, Poland) according to the manufacture protocol. For qPCR analysis, samples of cDNA were run in triplicates using a dilution 1:10 and carried out using SYBER Green Master Mix (Applied Biosystem, Foster city, CA, USA), for this purpose we are using a sequence-specific primers whit the following sequences (Table 2).

### 4.11. Immunocytochemistry

Cells were seeded over cell imaging cover glasses (Eppendorf). After the treatments, cells were washed in PBS and fixed in 4% paraformaldehyde in 0.1M phosphate buffer pH: 7.4 for 10 min at room temperature (RT). After fixation, cells were washed thrice in PBS, permeabilized and blocked with a solution containing 0.15% Tween-20 and 0.5% BSA for 30 min at RT. Once blocking solution was removed, and specific monoclonal antibody anti-α-tubulin (1:200) or mycotoxin phalloidin (1:500) was added and incubated overnight at 4 °C in a humid chamber. The next day, cells were washed in PBS and incubated with Goat anti-mouse fluorescent secondary antibody (Alexa Fluor 488, 1:500) for 1 h at RT. After incubation, cells were washed in PBS and the nuclei were stained with 4’, 6-diamino-2-phenylindole (DAPI) at a concentration of 1 μg/mL for 5 min at RT. Finally, the culture chamber was removed, and slides were mounted using Fluoromont G (Dako, Produktionsvej, Glostrup, Denmark). For the estimation of the globular (G): polymerized actin (F) ratio we carried out a protocol based on Deoxyribonuclease I kit (Invitrogen, Carlsbad, CA, USA) in combination with phalloidin staining. 

### 4.12. Quantification of Mitosis Number

To calculate the number of mitosis present in the cell culture after treatment with melatonin, DAPI images from immunocytochemistry were used and the ratio between the number of mitosis respect to the total number of cells was calculated, analyzing a total of 18 fields per experimental group.

### 4.13. Cell Volume Analysis

To calculate the in situ cell volume in cultured cells, we used a protocol previously developed by our research group, with minor modifications. To this aim, confocal microscopy micrographs of phalloidin staining samples, taken at a final 200× magnifications, were processed using the Imaris image processing software (Bitplane, Oxford instruments, Belfast, UK).

### 4.14. Animal Models

All the experiments were designed with the approval (07/15/2016) of the ethical committee on animal experimentation of the University of Oviedo (PROAE 32/2016). Likewise, surgical and treatment procedures applied were carried out following the European Directive 2012/63/EU. To carry out the experiments, a cohort of 24, 10 week-old mice (*Mus musculus*) on a C57BL/6J genetic background were acquired from Charles River (Wilmington, MA, USA). This genetic background was chosen given the inability of these animals to produce melatonin, since they present a truncated form of the enzyme aril-alkylamine-N-Acetyltransferase (AANAT), the key enzyme in the synthesis of indole. All the animals were identified, housed individually and maintained under a 12:12 light dark cycle with food and water provided ad libitum. Lung metastasis from melanoma cells was induced by injection of B16-F10 cells in the tail vein, using a well-reported protocol. In all the experiments, animals were sacrificed 15 days after injection. Blood was collected by cardiac puncture and then part of both lungs were dissected and fixed overnight in a solution containing paraformaldehyde 4% in phosphate buffer 0.1M, pH 7.4. Subsequently, tissues were embedded in paraffin and 5 μm sections were obtained and stained with hematoxylin/eosin. Additionally, metastasis present in the lungs were isolated by microsurgery and frozen in liquid nitrogen to perform molecular biology studies.

### 4.15. Injection of Cells

When B16-F10 cells were confluent, they were trypsinized following the method described above. Cells were then counted in a hemocytometer and resuspended in DMEM medium without supplements at a final concentration of 10^6^ cells/mL. From this suspension, 2 × 10^5^ cells were injected into the tail vein of each mouse.

### 4.16. In Vivo Luciferase Imaging

Bioluminescence imaging was performed with an IVIS imaging system (Xenogen Corp, Alameda, CA, USA). After 10 days of cell injection, mice were IP injected with D-Luciferin potassium salt (Perkin Elmer, MA, USA) at 150 mg/Kg body weight, under isoflurane anesthesia (Zoetis, NJ, USA). Luminescence was measured 5 min after the injection, taking anatomical images in Dorsal, Lateral and Ventral position of each animal.

### 4.17. Macroscopic and Microscopic Quantification of Pulmonary Metastasis

For the quantification of the surface occupied by melanoma metastasis in relation to the pulmonary surface, two macros were designed for the free image J software. These tools allow an automatic analysis of the surface of each of the metastasis, as well as the surface occupied by lung tissue both macroscopically and microscopically.

### 4.18. Tumor Model with Intraperitoneal Melatonin (IP)

To perform the melanoma tumor model in vivo, animal cohort was divided into two experimental groups. For visualization four animals of both experimental groups received the injection of luciferase-transformed B16-F10 cells, which were kindly provided by Dr. López-Otín. The other group of 20 animals were injected with parental B16-F10 cells. Both groups were equally divided into treatment and control groups. Treated group were administered melatonin daily at a dose of 20 mg/kg body weight by intraperitoneal (IP) injection was performed two hours before the dark phase. Control group was administered a vehicle (10% ethanol). 

### 4.19. Tumor Model with Melatonin in Drinking Water

For the realization of the melanoma tumor model in vivo another cohort of 16 animals C57BL/6J was equally divided into two groups to which B16-F10 cells transformed with luciferin were administered. One of the groups was administered melatonin at a dose of 20 mg/kg of body weight in the drinking water, while the control group was administered vehicle (10% ethanol). The amount of water consumed was recorded every two days and animals were sacrificed 15 days after the injection of the cells.

## 5. Conclusions

In view of these results, we can conclude that melatonin meets an antitumor papal in the in vitro model of melanoma B16-F10 carried out for its antiproliferative action, reducing the growth rate of cells and reducing their ability to migrate, which seems to be directly related to the presence of indole and its receptor-mediated effect, which would trigger an alteration of the cell cycle and cell cytoskeleton, not happening in murine models.

## Figures and Tables

**Table 1:** Anti-human antibodies employed for Western blot (WB), immunocytochemistry (ICC) and immunohistochemistry (IHC).

Antibody	Company	Reference	Lot#	(Conc.)
Catalase (CAT)	Calbiochem	219010	A00056904	1:5000
SOD2/MnSOD	Millipore	06-984	2557606	1:5000
Thioredoxin (TRX1)	YMCO	ATRX-06		1:1000
CDK-1 (A-17)	Abcam	Ab18	GR3186726-2	1:1000
Cyclin B1 (EPR170609)	Abcam	Ab181593	GR238750-21	1:2000
β-Actin (AC-15) (ACTB1)	Santa Cruz	SC-69879	C2818	1:8000
α-Tubulin (B-5-1-2)	Santa Cruz	SC-23948	B0212	1:1000
Anti-mouse	Millipore	12-349	2722855	
Anti-rabbit	Millipore	12-348	3166073	

**Table 2:** Primers sequences used for the indicated murine genes.

Gen	Forward Primer	Reverse Primer
β-Actin	5′-GGCTGTATACCCCTCCAT-3’	5′-CCAGTTGGTAACAATGCCATG-3′
α-Tubulin	5′-TCGATATTGAGCGTCCAACCT-3′	5′-CAAAGGCAACAATGCCATGT-3′

## Supplementary Materials

Supplementary materials can be found at https://www.mdpi.com/1422-0067/21/2/548/s1.
