# Supplementary material for: Melatonin-Induced Cytoskeleton Reorganization Leads to Inhibition of Melanoma Cancer Cell Proliferation
Source: Int J Mol Sci. 2020 Jan 15;21(2):548. doi: 10.3390/ijms21020548 (PMC7014391; doi:10.3390/ijms21020548)
Supplement: Supplementary file 1 [file ijms-21-00548-s001.pdf]

International Journal of
Molecular Sciences

MDPI

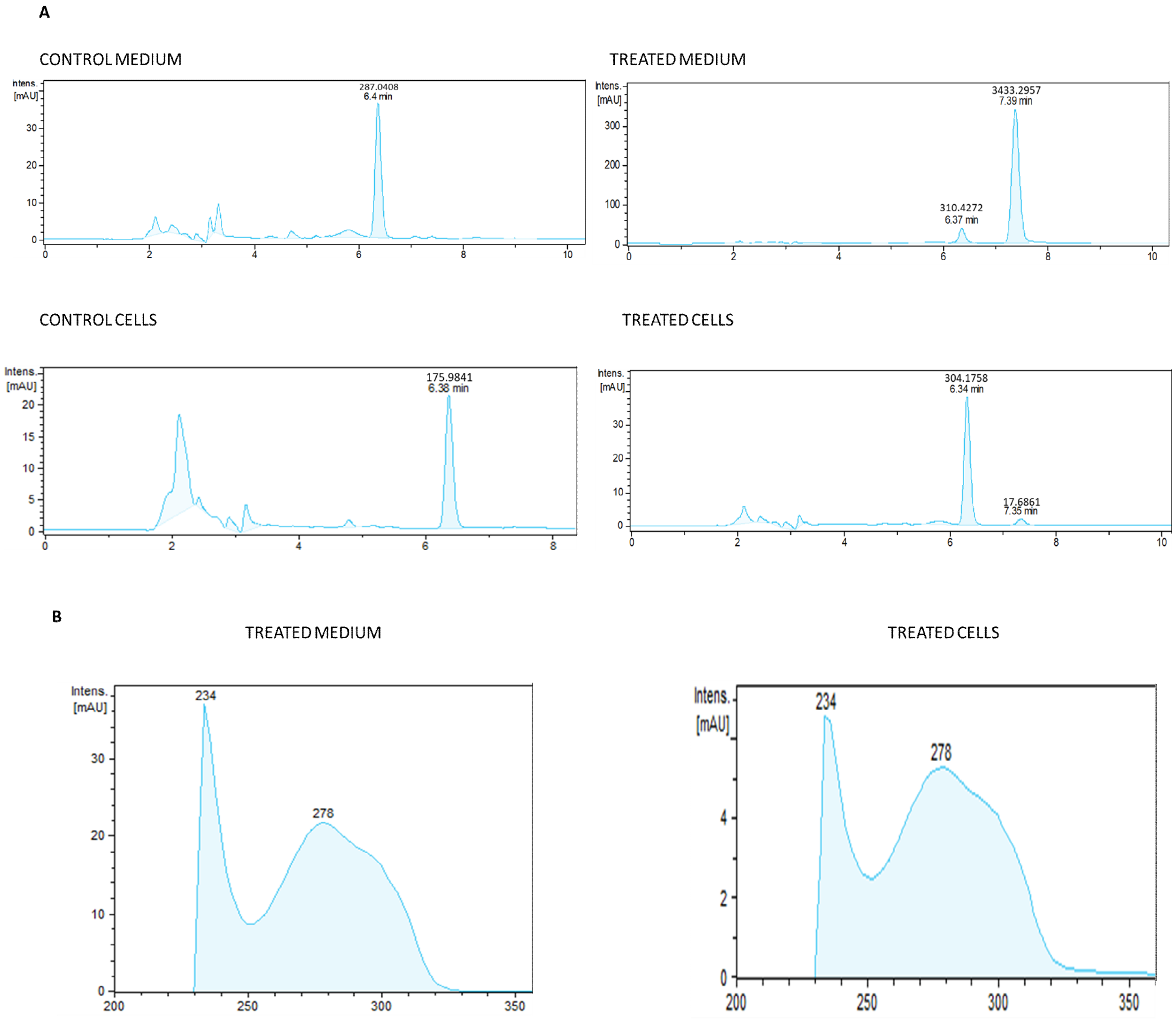

Supplementary Figure S1. Melatonin detection by HPLC in B16-F10 cells and culture medium. (A) Chromatogram of Control Medium and Control Cells and respective treated groups. (B) Absorption spectra of treated medium and cells.

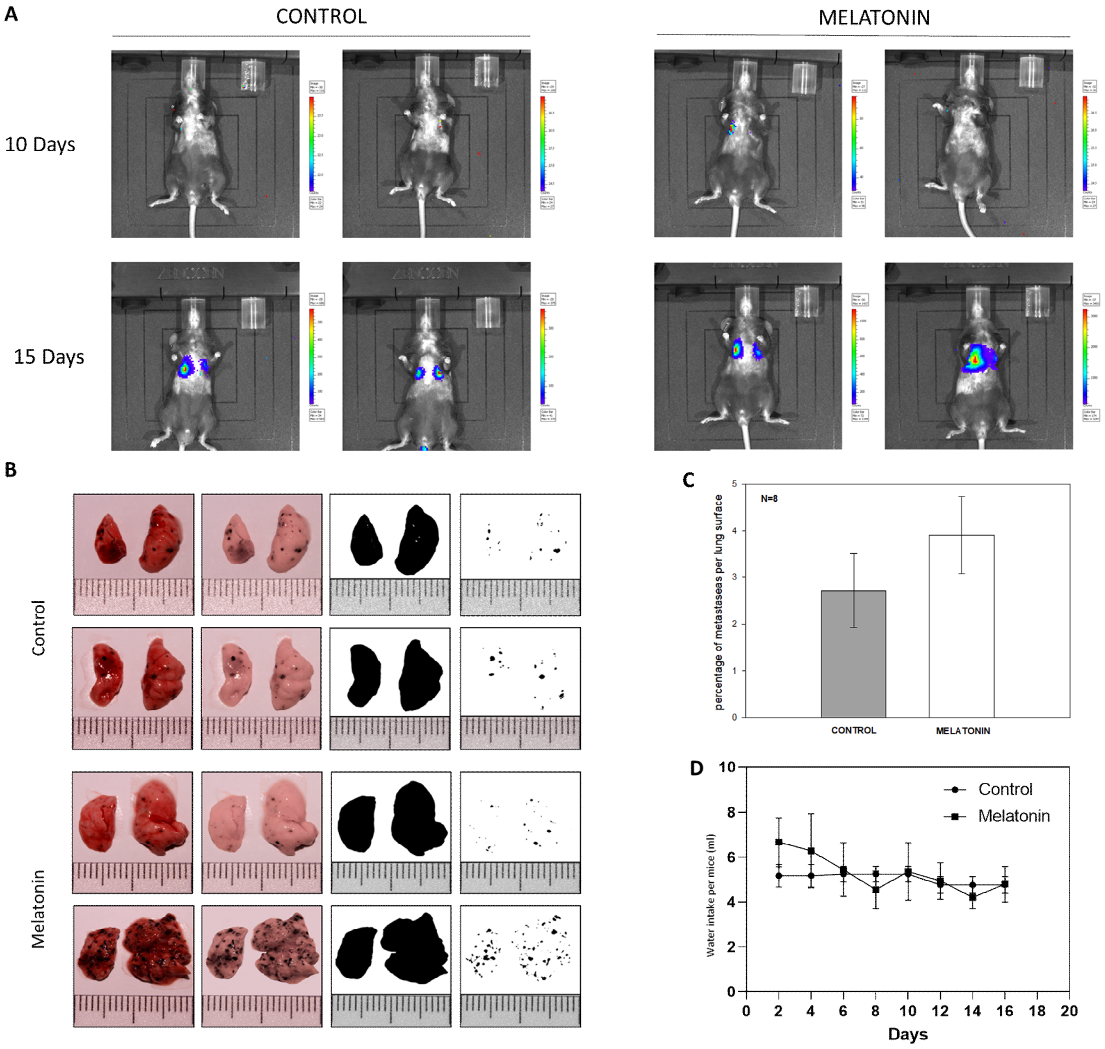

Supplementary figure S2. ***In vivo*** **model for B16-F10 lung metastases treated with drink water Melatonin.** (A) Bioluminiscence, localization and size of total lung metastaseas. (B) Macroscopic sections of lung metastaseas and image isolation process. (C) Percentage of metastaseas Surface per total lung Surface. (D) Water intake per mice. Data were shown as average +/- SEM.
